# SIK2 activates the autophagy‒apoptosis pathway through SP1 regulation to inhibit the progression of hepatocellular carcinoma

**DOI:** 10.3389/fphar.2025.1635953

**Published:** 2025-10-02

**Authors:** Sheng Fan, Yan Zhang, Pengcheng Ma, Huanan Hou, Ruiqi Niu, Ziming Wang, Jinguo Zhang, Yunhong Xia, Yueyin Pan

**Affiliations:** ^1^ Department of Oncology, The First Affiliated Hospital of Anhui Medical University, Hefei, Anhui, China; ^2^ Anhui Public Health Clinical Center, Hefei, Anhui, China; ^3^ Department of Cardiology, The First Affiliated Hospital of Anhui Medical University, Hefei, Anhui, China; ^4^ Department of General Surgery, The First Affiliated Hospital of Anhui Medical University, Hefei, Anhui, China; ^5^ Department of Medical Oncology, The First Affiliated Hospital of USTC, Division of Life Sciences and Medicine, University of Science and Technology of China, Hefei, China

**Keywords:** hepatocellular carcinoma, SIK2, autophagy, apoptosis, signaling pathway, tumor suppression

## Abstract

Hepatocellular carcinoma (HCC) ranks as the sixth most prevalent cancer globally and is the fourth leading cause of cancer-related mortality, characterized by limited treatment options and an unfavorable prognosis. Salt-inducible kinase 2 (SIK2), a member of the AMP-activated protein kinase (AMPK) family, regulates cellular processes, including metabolism, autophagy, and apoptosis. However, its specific role in HCC remains unclear. This study assessed the clinical relevance and biological function of SIK2 in HCC via bioinformatics, immunohistochemistry (IHC), cell assays, signaling pathway analyses, and animal models. The results demonstrated that high SIK2 expression was associated with improved patient survival, modulation of the immune microenvironment, and suppression of tumor progression. Mechanistically, SIK2 inhibited HCC cell proliferation, migration, and invasion and promoted autophagy through increased autophagic flux. However, due to impaired autophagic flux, apoptosis is induced. This study highlights the significant clinical relevance of SIK2 in primary liver cancer and its multifaceted roles in tumor biology. SIK2 serves as an independent protective prognostic factor and may exert a tumor-suppressive effect by modulating the tumor microenvironment, autophagy, and apoptosis. Elevated SIK2 expression was strongly linked to better prognosis in HCC patients, highlighting its promise as both a prognostic indicator and a potential therapeutic target. Future research should focus on clarifying the precise molecular mechanisms involving SIK2 and investigating its potential for clinical therapeutic applications.

## 1 Introduction

Hepatocellular carcinoma (HCC) ranks among the most prevalent malignant tumors worldwide and is characterized by high incidence and mortality, with a notably high occurrence in Asia (Siegel et al., 2023; Bray et al., 2024). Despite advancements in early detection, surgical interventions, and targeted therapies for liver cancer in recent years, the prognosis for patients with HCC remains unfavorable owing to its intricate pathological mechanisms. Clinical treatment faces challenges such as drug resistance, recurrence, and metastasis (Llovet et al., 2021; Vogel et al., 2022). To gain deeper insight into the progression of HCC and identify novel therapeutic targets, investigating liver cancer-associated genes and their regulatory pathways has increased in importance.

Among the various possible regulatory mechanisms, autophagy and apoptosis are key determinants of liver cancer cell growth and survival (Schwabe and Luedde, 2018; Debnath et al., 2023). Autophagy, as a self-clearing mechanism of cells, helps maintain cellular homeostasis by degrading damaged organelles and proteins (Mizushima and Komatsu, 2011). However, both excessive and insufficient autophagy can affect tumorigenesis and progression (Li et al., 2020; Mohammed et al., 2024). Apoptosis represents a primary mode of cell death, and its suppression in tumor cells frequently contributes to drug resistance and disease recurrence (Koren and Fuchs, 2021). Autophagy and apoptosis, as core processes in the regulation of cellular homeostasis, maintain a dynamic balance during tumor progression (González-Polo et al., 2005; Sorice, 2022). Previous studies have shown that autophagy and apoptosis form an interactive network through shared signaling pathways (such as the PI3K/AKT/mTOR and MAPK/ERK pathways), cross-talk in various forms and actions, and affect tumor cell proliferation, metastasis, and treatment response (Mariño et al., 2014). In HCC, abnormal expression of autophagy-related genes (such as LC3B and p62) is associated with patient prognosis, whereas dysregulation of the apoptosis pathway (such as the caspase cascade) may affect disease progression (Young et al., 2012). Studying the relationship between autophagy and apoptosis in HCC helps further understand the mechanism of liver cancer development. Uncovering their functions in liver cancer may offer novel insights for early diagnosis and therapeutic intervention.

Salt-inducible kinase 2 (SIK2), a significant member of the AMP-activated protein kinase (AMPK) family, is intimately involved in key biological processes such as cellular energy regulation, autophagy, and programmed cell death (Du et al., 2016a). As a member of the AMPK family, SIK2 is activated by phosphorylation through liver kinase B1 (LKB1), also known as STK11 (a serine/threonine kinase), which then activates a series of downstream signaling pathways (Lizcano et al., 2004). In recent years, there has been increasing research on the expression and function of SIK2 in various cancers, with studies indicating that SIK2 may have dual roles in promoting or inhibiting cancer in different tumors. Notably, in a study by Li *et al.*, SIK2 was found to be downregulated in liver cancer, where it exhibited tumor-suppressive effects. However, its role in liver cancer remains unclear, and its function has not been systematically elucidated (Du et al., 2016b; Sun et al., 2020). Preliminary bioinformatics analysis in this study suggested that SIK2 expression levels are correlated with survival and immune microenvironment scores in HCC patients, but it remains unclear whether SIK2 influences tumor progression through the regulation of the interaction between autophagy and apoptosis. Given the potential role of the autophagy‒apoptosis imbalance in HCC drug resistance and recurrence, understanding the regulatory mechanisms of SIK2 in this process may provide new insights for therapeutic strategies (Amir et al., 2013; Han et al., 2021).

This study focused on the multidimensional functions of SIK2 in HCC, combined clinical data analysis with experimental validation, and systematically explored its clinical significance and molecular mechanisms. These findings provide new theoretical evidence for the prognostic assessment and targeted therapy of liver cancer. Future research could further explore the synergistic effects of SIK2 with existing therapies and develop combination treatment strategies on the basis of its regulatory pathways, with the aim of providing scientific support to improve the clinical prognosis of HCC patients.

## 2 Materials and methods

### 2.1 Data processing

SIK2 mRNA expression profiles in HCC and adjacent normal liver tissues were acquired from The Cancer Genome Atlas (TCGA) database (https://portal.gdc.cancer.gov/). This dataset comprises 424 clinical specimens with annotated clinicopathological characteristics and survival outcomes. Primary data curation and preprocessing were conducted via Strawberry Perl (v5.30.0.1), followed by advanced statistical analyses implemented through the R programming language (v4.1.3).

### 2.2 Bioinformatics

To elucidate the functional relevance of SIK2 in hepatocellular carcinogenesis, integrative bioinformatics approaches were applied to TCGA-LIHC cohorts. Survival correlation analyses, which quantify the prognostic significance of SIK2 transcriptional levels in HCC patients, were initially derived from the TCGA database and subsequently cross-validated via immunohistochemical evidence archived in the Human Protein Atlas (https://www.proteinatlas.org). R language-based analyses included clinical correlation analysis, independent prognostic assessment, tumor microenvironment (TME) heterogeneity profiling, immune cell infiltration quantification, immunotherapy response prediction, SIK2 co-expression analysis, Kyoto Encyclopedia of Genes and Genomes (KEGG) pathway enrichment analysis, autophagy-related gene correlation analysis, and pharmacogenomic sensitivity evaluation.

SIK2 target genes were predicted via four databases: HumanTFDB (http://bioinfo.life.hust.edu.cn/HumanTFDB), GTRD (https://gtrd20-06.biouml.org), PROMO (https://alggen.lsi.upc.es/cgi-bin/promo_v3/promo/promoinit.cgi?dirDB=TF_8.3), and the UCSC Genome Browser (http://genome.ucsc.edu) integrated with the JASPAR database (https://jaspar.genereg.net). Overlapping targets were systematically identified via the “VennDiagram” R package and cross-referenced with SIK2-associated co-expressed genes. Transcriptional regulatory elements in the SIK2 promoter were investigated via the JASPAR database’s TF-binding prediction algorithms.

### 2.3 Patient and sample collection

This investigation analyzed 118 paired HCC specimens with adjacent nonmalignant tissues procured from the First Affiliated Hospital of Anhui Medical University. The eligibility requirements are as follows: confirmed HCC diagnosis through postoperative histopathology, absence of preoperative anticancer therapies, and accessibility of comprehensive clinicopathological documentation.

### 2.4 IHC

Immunohistochemical analysis was conducted to evaluate SIK2 protein localization in HCC specimens. The experimental workflow comprised (1) tissue fixation in 10% neutral buffered formalin followed by ethanol gradient dehydration and paraffin embedding; (2) sectioning at a thickness of 4 μm with subsequent oven heating (90 C, 30 min) and sequential dewaxing with xylene/ethanol solutions; and (3) microwave-mediated antigen retrieval under dual-phase conditions (full power for 2.5 min → 30% power for 8 min). Postretrieval processing included three PBS washes (Biosharp, China), endogenous peroxidase inactivation with 3% H_2_O_2_, and serum blocking with 3% BSA (Biosharp, China). Primary antibody incubation proceeded at 4 C for 16 ± 2 h, followed by incubation with an HRP-conjugated secondary antibody (1:100 dilution) at room temperature for 120 min. Chromogenic development was performed with DAB substrate (5–10 min reaction monitoring), which was terminated by immersion in distilled water. Counterstaining with Mayer’s hematoxylin preceded final dehydration and neutral balsam mounting. The staining intensity was scored from 0 to 3 on the basis of color intensity (none, weak, moderate, or strong); the area was scored from 0 to 4 according to the percentage range (0%, 1%–25%, 26%–50%, 51%–75%, or >76%). The results were excluded for tissue detachment or nonspecific staining.

### 2.5 Cell culture and transfectionj

The LO2 and THLE2 nonmalignant human hepatic epithelial cell lines, HCC cell lines (Hep3B, HuH-7, MHCC97H, LM3), and HEK293T cells were sourced from either the Shanghai Cell Bank Type Culture Collection Committee or the American Type Culture Collection (ATCC). All the cell lines were maintained in Dulbecco’s modified Eagle’s medium (DMEM; 4.5 g/L D-glucose, Gibco, USA) supplemented with 10% fetal bovine serum (FBS; Gibco, USA), 100 U/mL penicillin and 100 μg/mL streptomycin (Biosharp, China). Standard culture conditions included incubation at 37 C under a 5% CO_2_ atmosphere.

In this study, the SIK2 overexpression plasmid was generated by Miaoling Biotechnology (Wuhan, China). SIK2-targeting siRNA and lentiviral particles were custom synthesized by GenePharma (Shanghai, China). Plasmid DNA was purified with an Endofree Plasmid Kit (Tiangen Biotech, DP118-02). Lentiviral vectors were generated by transiently transfecting HEK293T cells with a triple-plasmid system comprising the transfer vector, packaging plasmids psPAX2 (Addgene, USA), and pMD2.G (Addgene, USA) using PEI transfection reagent (Polysciences, USA). Genetically modified cellular clones were generated via puromycin-based antibiotic selection (2 μg/mL, 72 h exposure), followed by validation of stable protein expression through immunoblotting assays. The corresponding target sequences are detailed in Supplementary Table S1.

### 2.6 Quantitative real-time PCR (qRT‒PCR)

RNA extraction from samples was achieved through RNAiso Plus reagent (Takara, Japan), and reverse transcription reactions were executed with the PrimeScript RT Master Mix Kit (Takara, Japan). RT‒qPCR amplification was performed on the LightCycler^®^ 480 II platform (Roche, Switzerland) with TB Green^®^ Premix Ex Taq™ (Tli RNaseH Plus) (Takara, Japan). The endogenous controls included β-actin and GAPDH. The PCR protocol involved an initial denaturation step at 95 C for 10 min, followed by 40 amplification cycles at 95 C for 15 s and 60 C for 1 min. Details of the primer sequences can be found in Supplementary Table S1.

### 2.7 Western blotting

Proteins separated by SDS‒PAGE (Beyotime, China) were transferred onto PVDF membranes (Millipore, USA). The membranes were blocked with 5% skim milk for 1.5 h at room temperature and then incubated overnight at 4 °C with primary antibodies, followed by a 1.5 h incubation at room temperature with horseradish peroxidase-conjugated secondary antibodies. Immunoreactive bands were detected via an enhanced chemiluminescence (ECL) substrate (Vazyme, China). Details regarding the antibody sources and working dilutions are provided in Supplementary Table S1.

### 2.8 Cell Counting Kit-8 (CCK-8) assay

Cellular proliferative activity was quantitatively analyzed via a Cell Counting Kit-8 (CCK-8) assay (Beyotime, China). Hep3B and HuH-7 cells were seeded into 96-well microplates at optimized densities and maintained under standard culture conditions for 24–96 h. At predetermined intervals, 10 µL of CCK-8 solution was added to each well containing 100 µL of culture medium, followed by incubation at 37 °C (5% CO_2_) for 60 min. Absorbance measurements were subsequently recorded at 450 nm using a multimode microplate reader (Thremo Fisher, USA).

### 2.9 Colony formation assay

For clonogenic survival assessment, cellular suspensions were plated at a density of 500 cells per well in 6-well culture plates and incubated under standard conditions (37 C, 5% CO_2_) for 14 days. Following the incubation period, colonies were fixed and stained with 0.1% crystal violet solution (Sigma‒Aldrich) to quantify their clonogenic potential.

### 2.10 Transwell assay

Cell migratory and invasive potentials were evaluated via 8-μm pore Transwell™ chambers (Corning, USA) in 24-well configurations. Hep3B and HuH-7 cells (5 × 10^4^ cells/well) suspended in serum-free DMEM were seeded into the upper chambers, with invasion assays requiring Matrigel-coated inserts (Corning, USA). The lower chambers contained 1,100 μL of DMEM (Gibco, USA) supplemented with 10% FBS (Gibco, USA). Following a 24 h culture period, the cell samples were fixed with 4% PFA (15 min treatment), followed by 30 min of exposure to 0.5% crystal violet. ImageJ software facilitated quantitative assessments through microscopic evaluation.

### 2.11 Wound healing assay

Hep3B and HuH-7 cells were plated in 6-well culture dishes at a density of 5 × 10^5^ cells/well and maintained until 90%–95% monolayer coverage was achieved. Uniform scratches were created via the use of a sterile 200 μL pipette tip aligned with a straight edge, followed by three washes with PBS and the addition of serum-free DMEM. Microscopic observation and imaging were performed at 0 h and 24 h postscratch. Migration rates (%) were calculated as [(A_0_ − A_24_)/A_0_] × 100 via ImageJ (v1.53, NIH), with experiments excluded for irregular edges or confluency below 80%.

### 2.12 Flow cytometric analysis of cell apoptosis

Cellular apoptosis in Hep3B and HuH-7 cells was quantified via a FITC Annexin V Apoptosis Detection Kit I (BD Biosciences, USA) following standard protocols. The detached cells were resuspended in 10 μL of 1× binding buffer and then costained with 5 μL of Annexin V-FITC and 5 μL of propidium iodide (PI). After 15 min of incubation in the dark at 25 C, fluorescence signals were acquired on a flow cytometer (Beckman, USA). Data analysis was executed via FlowJo software (v10.81, FlowJo LLC).

### 2.13 Autophagic flux counting

Cellular transduction was performed via the AdPlus-mCherry-GFP-LC3B adenoviral construct (Beyotime, China) following the manufacturer’s protocol. After transduction, the target cells demonstrated robust expression of the tripartite fusion protein comprising mCherry (red fluorescence), GFP (green fluorescence), and the autophagy marker LC3B. Fluorescence microscopy revealed the cytoplasmic distribution of the probe under basal conditions, manifesting as homogeneous yellow emission (merged GFP/mCherry signals). Autophagic induction triggered the selective recruitment of mCherry-GFP-LC3B to autophagosomal membranes, which were visualized as distinct yellow punctate structures. Subsequent autophagosome‒lysosome fusion events led to pH-dependent attenuation of GFP fluorescence within acidic lysosomal compartments, yielding residual red punctiform signals indicative of mCherry retention.

### 2.14 Chromatin immunoprecipitation-quantitative polymerase chain reaction (ChIP‒qPCR)

Chromatin immunoprecipitation (ChIP) assays were conducted via a ChIP Assay Kit (Cell Signaling Technology, USA) according to the manufacturer’s protocol. Lysates containing soluble chromatin were incubated overnight with anti-SP1 antibody (Proteintech, China), histone H3, or normal human IgG as an isotype control for immunoprecipitation. Post-IP purification was achieved through spin column chromatography. Decrosslinked DNA templates were PCR-amplified to target human SIK2 genomic regions. The quantitative data represent five independent experimental replicates.

### 2.15 Luciferase reporter assay

The full-length and mutant SIK2 promoter sequences were subsequently cloned and inserted into pGL3-Basic vectors (MluI/XhoI digestion, PCR validation, and sequencing confirmed by Wuhan Servicebio Biotechnology). HEK-293T cells seeded in 6-well plates (80% confluency) were cotransfected with 2.5 μg of SP1 plasmid (control/overexpression), 0.25 μg of pRL-TK, or 7.5 μg of reporter plasmid (wild-type/mutant/empty vector) via EZ Trans Plus reagent. Eight hours following transfection, the culture medium was changed. After 48 h, the cell lysates were prepared by treating the cells with 500 μL of lysis buffer for 10 min at room temperature, followed by centrifugation at 12,000 × g for 10 min at 4 C. Firefly and Renilla luciferase activities were quantified via a microplate reader. Activity ratios (firefly/Renilla) were calculated to normalize the transfection efficiency.

### 2.16 Mouse xenograft model

HuH-7 cells (vector or OE-SIK2; 5 × 10^6^ cells suspended in 200 μL of PBS) following 48 h of lentiviral transduction were subcutaneously implanted into the right lower flank of male BALB/c nude mice (5 weeks old; n = 5/group). Tumor growth kinetics were monitored at 3-day intervals via Vernier calipers, and volumetric calculations were performed via the following formula: *V* = length × width^2^ × 0.5. On day 30 postinoculation, the mice were euthanized, and the tumors were excised, weighed, and photographed. Resected tissues were either fixed in 4% PFA or flash-frozen in liquid nitrogen for subsequent analytical procedures.

### 2.17 Statistical analysis

Data processing and statistical analyses were conducted via GraphPad Prism 9, IBM SPSS Statistics 27, and R (v4.1.3). All experimental findings were derived from a minimum of three independent replicates. For statistical evaluations, two-group comparisons were performed via two-tailed unpaired t tests, whereas for multigroup comparisons, one-way or two-way ANOVA was performed via GraphPad Prism 9. The graphical data are presented as error bars denoting mean ± SD/SEM. Significance levels are annotated as follows: ns (not significant), *P < 0.05, **P < 0.01, ***P < 0.001, and ****P < 0.0001.

## 3 Results

### 3.1 Multidimensional bioinformatics analysis of SIK2 in HCC

**FIGURE 1:**
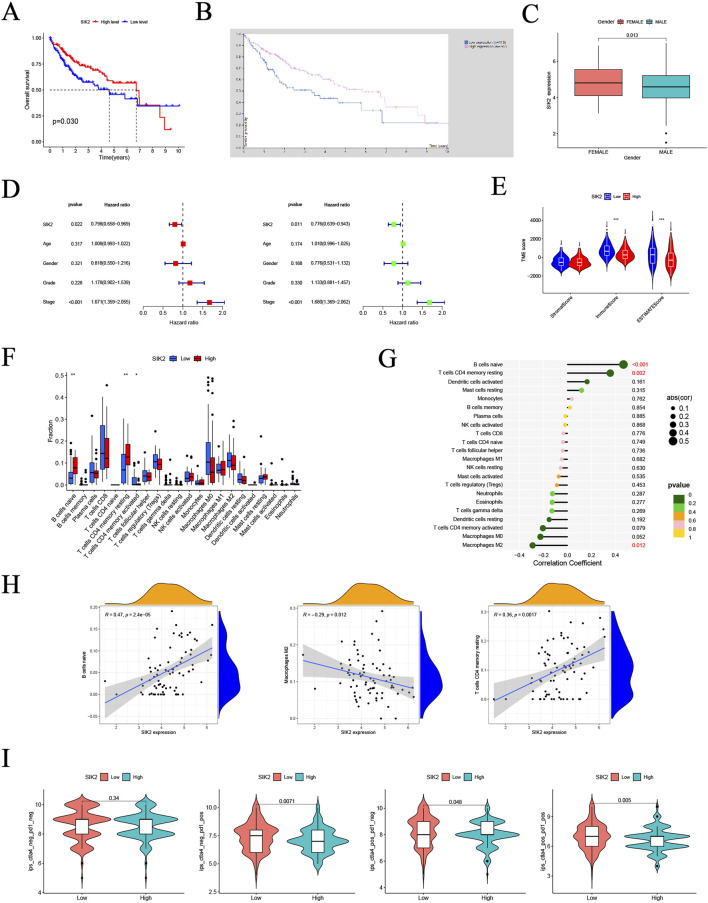
Multi-dimensional bioinformatics analysis of SIK2 in HCC. **(A,B)** Kaplan-Meier overall survival (OS) curves for SIK2 in HCC from TCGA and THPA. **(C)** Association between SIK2 expression and gender in HCC. **(D)** Univariate and multivariate Cox regression identified SIK2 as an independent OS predictor. **(E)** Sample distribution of high/low SIK2 groups across different tumor microenvironment (TME) scores. **(F)** Infiltration of Naive B cells, resting memory CD4^+^ T cells was upregulated, while activated memory CD4^+^ T cells was downregulated in high SIK2 group. **(G)** Lollipop plot: Naive B cells positively correlated, M2 macrophages negatively correlated with SIK2. **(H)** TIMER scatter plots: SIK2 positively correlated with Naive B cells, resting memory CD4^+^ T cells, and negatively with M2 macrophages. **(I)** Immune therapy scores of anti-CTLA4/anti-PD1 inhibitors between high/low SIK2 groups.

To investigate the associations between SIK2 and clinical characteristics, we analyzed the TCGA-LIHC dataset and performed integrated bioinformatic analyses with Kaplan‒Meier survival curves generated from the HPA platform. The results demonstrated that patients with low SIK2 expression had significantly shorter overall survival (OS) than did those with high SIK2 expression (Figures 1A,B). Correlation analyses between SIK2 expression and clinical parameters (age, sex, tumor grade, and stage) revealed that female HCC patients exhibited significantly higher SIK2 expression levels than male patients did (*P* < 0.05), while no significant correlations were observed with other clinical features (Figure 1C). Univariate and multivariate Cox regression analyses were subsequently performed to evaluate the independent prognostic value of SIK2 in HCC. SIK2 emerged as a significant independent protective prognostic factor (univariate analysis: HR = 0.798, 95% CI: 0.658–0.969, *P* = 0.022; multivariate analysis: HR = 0.776, 95% CI: 0.639–0.943, *P* = 0.011). In contrast, tumor stage was identified as a significant risk factor (univariate analysis: HR = 1.671, 95% CI: 1.359–2.055, *P* < 0.001; multivariate analysis: HR = 1.680, 95% CI: 1.369–2.062, *P* < 0.001). Age, sex, and tumor grade did not reach statistical significance in either analysis (*P* > 0.05) (Figure 1D).

To further elucidate the biological mechanisms of SIK2, we investigated its association with the TME. We analyzed differences in TME scoring metrics between the high- and low-SIK2 expression groups. Significant differences were observed in immune scores and ESTIMATES scores between the high and low SIK2 expression groups, whereas no significant difference was found in stromal scores between the two groups (Figure 1E). Furthermore, correlation analyses between SIK2 expression levels and immune cell infiltration revealed that high SIK2 expression was positively correlated with the infiltration levels of antitumor immune cells (e.g., naive B cells and resting memory CD4^+^ T cells) and negatively correlated with immunosuppressive M2 macrophages (Figures 1F–H).

Building upon the findings from immune infiltration analyses, we further investigated the impact of SIK2 expression levels on immunotherapy efficacy in HCC patients. Using immunophenoscore (IPS) data from the TCIA database (N = 371 LIHC cases), our results demonstrated that high SIK2 expression was significantly associated with higher IPS values with multiple immunotherapy combinations, particularly under PD-1-positive conditions (*P* = 0.0071 and *P* = 0.005). In contrast, no significant effect of SIK2 expression on treatment efficacy was observed when both PD-1 and CTLA-4 were negative (*P* = 0.34) (Figure 1I).

### 3.2 Correlations between SIK2 expression and clinical characteristics with prognostic evaluation in 118 HCC patients

**FIGURE 2:**
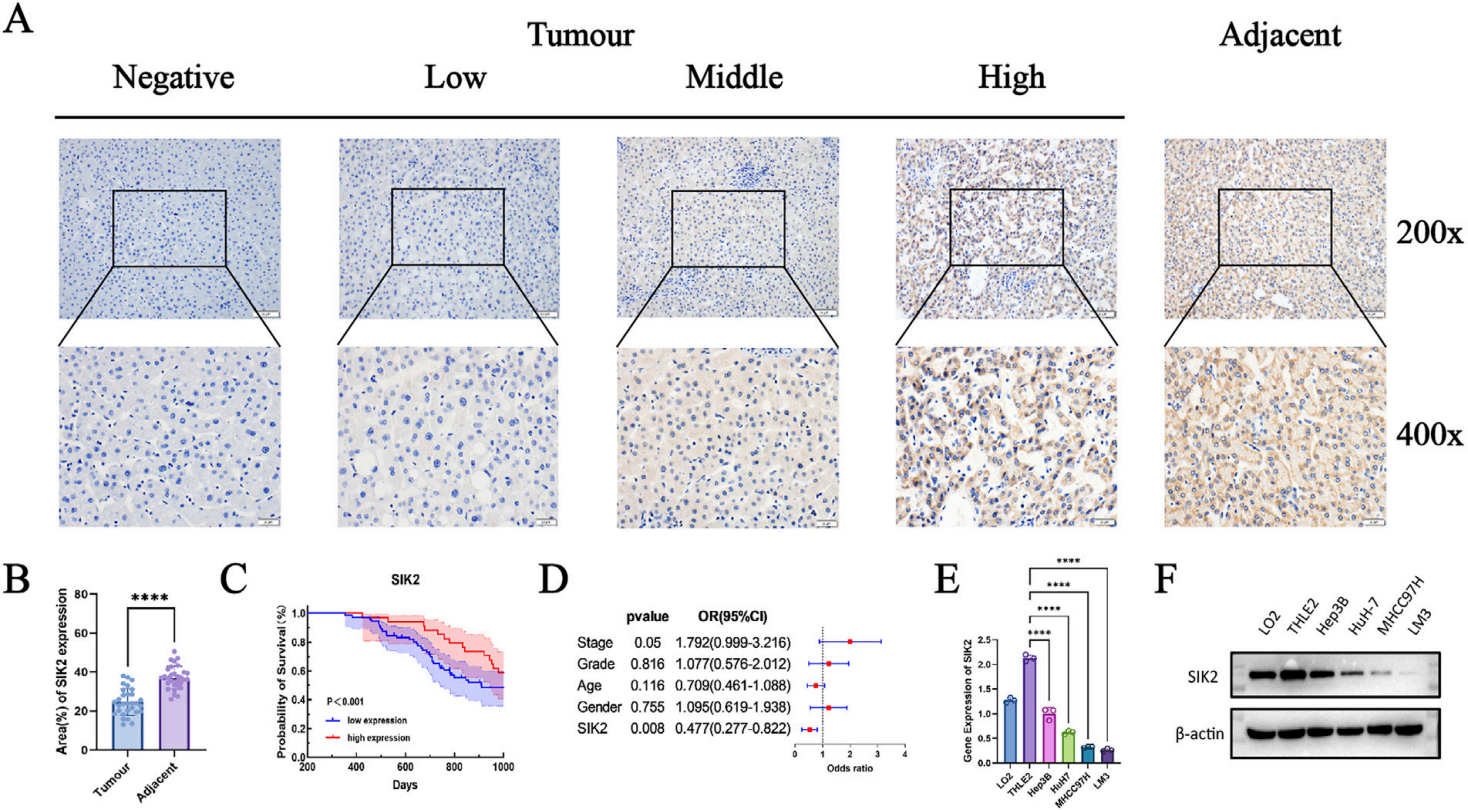
SIK2 was downregulated in HCC and correlated with a better prognosis. **(A)** Immunohistochemical (IHC) staining of SIK2 in adjacent non-tumor and tumor tissues. **(B)** SIK2 is highly expressed in adjacent tissues but reduced in HCC tumors. **(C,D)** Survival and multivariate Cox analysis of SIK2 in 118 HCC patients (analyzed by IBM SPSS, visualized by GraphPad Prism). **(E,F)** SIK2 transcription (RT-qPCR, ACTB as control) and protein (Western blot, β-actin as control) in HCC cell lines. Data: mean ± SD/SEM (n = 3); analyzed by two-tailed unpaired t-test or ANOVA. *P < 0.05, **P < 0.01, ***P < 0.001, ****P < 0.0001.

To further validate the results of the bioinformatics analysis and investigate the expression of SIK2 in HCC, we conducted immunohistochemical analysis and clinical cohort studies on 118 HCC patients and their pathological tissues. First, immunohistochemical analysis was performed on tumor tissues and adjacent paracancerous tissues from all 118 patients. In tumor tissues, the majority of samples exhibited mild or no staining, whereas in adjacent paracancerous tissues, most samples presented a deep brown coloration. These findings indicate that SIK2 expression levels are significantly lower in tumor tissues than in adjacent paracancerous tissues and that SIK2 expression is primarily localized in the cytoplasm (Figure 2A). Statistical analysis of the immunohistochemical results revealed that the immune scores of SIK2 in tumor tissues were significantly lower than those in adjacent paracancerous tissues (Figure 2B).

In further clinical cohort studies, we found that SIK2 expression levels were significantly correlated with patient sex and recurrence status (*P* = 0.0175 and *P* = 0.0013, respectively) but not with age, pathological grade, or tumor stage (*P* > 0.05). The proportion of high SIK2 expression in male patients was significantly lower than that in female patients, with the risk of high expression in females being 3.38 times greater than that in males. In recurrent patients, the high expression rate of SIK2 was only 16.9%, which was significantly lower than the 45.8% reported in nonrecurrent patients, with high-expression patients showing a 76% reduction in recurrence risk (Table 1). Subsequent survival analysis demonstrated that patients with high SIK2 expression had better survival outcomes, with significantly higher survival probabilities than those with low SIK2 expression (*P* < 0.001) (Figure 2C). Furthermore, forest plot analysis further confirmed the potential of SIK2 as a prognostic factor for evaluating outcomes in HCC patients (Figure 2D).

**TABLE 1:** Association Between SIK2 Expression Levels (Low vs High) and Clinical Characteristics and Prognosis in Patients.

Clinical characteristics	LIHC(N = 90)	SIK2 expression		*P value*
		Low (N = 81)	High (N = 37)	
Gender				0.0175
Male	98	72	26	
Female	20	9	11	
Recurrence				0.0013
YES	59	49	10	
NO	59	32	27	
Death				0.3241
YES	94	63	32	
No	23	18	5	
Age (years)				0.5483
<60	51	37	14	
≥60	67	44	23	
Grade				0.4256
G1+G2	53	34	19	
G3+G4	65	47	18	
Stage (T)				0.553
T1	58	38	20	
T2-T3	60	43	17	

Moreover, to investigate the differential expression of SIK2 across HCC cell lines, we performed qRT‒PCR and Western blot analyses. The results revealed that SIK2 was highly expressed in normal hepatocyte cell lines (LO2 and THLE2) but was notably reduced in HCC cell lines (Hep3B, HuH-7, MHCC97H, and LM3) (Figures 2E,F), which was consistent with previous immunohistochemical findings.

### 3.3 SIK2 functions as a tumor suppressor in HCC

**FIGURE 3:**
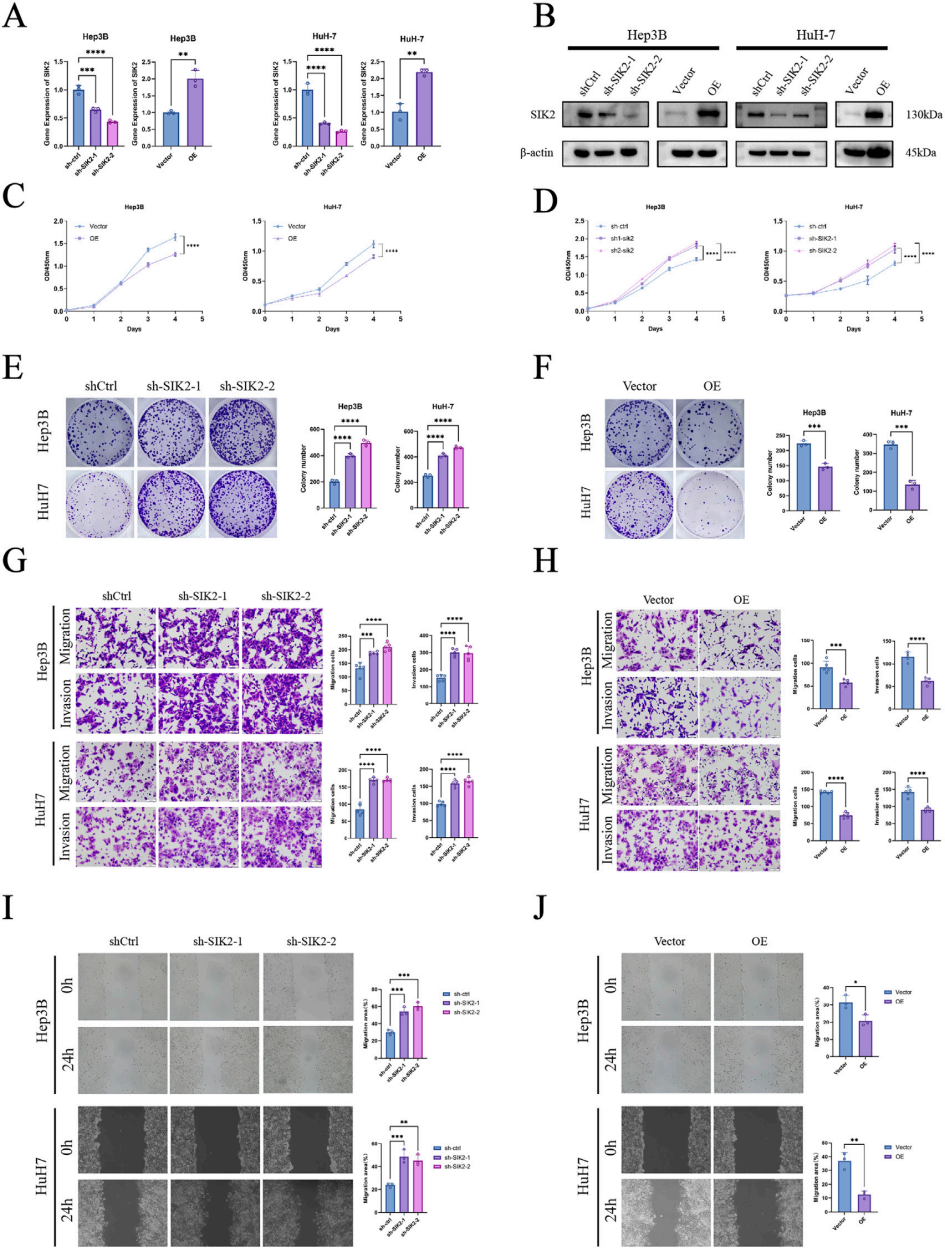
SIK2 inhibited the progression of HCC *in vitro*. **(A,B)** SIK2 knockdown or overexpression in Hep3B and HuH-7 via lentivirus (Western blot: β-actin as control; RT-qPCR: ACTB as control). **(C–F)** Proliferation assessed by CCK-8 and colony formation (quantified by ImageJ). **(G,H)** Migration and invasion via Transwell (quantified by ImageJ). **(I,J)** Migration via wound healing (quantified by ImageJ). Data: mean ± SD/SEM (n = 3); analyzed by two-tailed unpaired t-test or ANOVA. *P < 0.05, **P < 0.01, ***P < 0.001, ****P < 0.0001.

To investigate the effects of SIK2 on HCC cells, we first constructed SIK2-overexpressing (OE) and SIK2-knockdown groups (sh-SIK2-1 and sh-SIK2-2) in Hep3B and HuH-7 cells via lentiviral transduction technology. The expression levels of SIK2 in the SIK2-overexpressing and SIK2-knockdown cells were validated by qPCR and Western blotting (Figures 3A,B). We assessed cell proliferation through CCK-8 assays and colony formation experiments. The results demonstrated that SIK2 knockdown significantly accelerated HCC cell proliferation and markedly enhanced colony-forming ability. Conversely, in the SIK2-overexpressing group, the proliferation rate of HCC cells was significantly slowed, and the colony-forming ability was drastically reduced (Figures 3C–F). These results suggest that SIK2 exerts tumor-suppressive effects by inhibiting cell proliferation.

To evaluate the impact of SIK2 on HCC cell migration and invasion, we conducted Transwell migration and invasion assays as well as wound healing experiments. Transwell assay results revealed that SIK2 knockdown significantly enhanced the migratory and invasive capabilities of Hep3B and HuH-7 cells, whereas SIK2 overexpression markedly suppressed these abilities. These findings indicate that SIK2 further inhibits the aggressiveness of HCC cells by restraining their migration and invasion (Figures 3G,H). The wound healing assay further confirmed that SIK2 overexpression significantly inhibited cell migration, whereas SIK2 knockdown substantially promoted migratory capacity (Figures 3I,J).

### 3.4 SIK2 overexpression promotes autophagic flux and induces apoptosis

**FIGURE 4:**
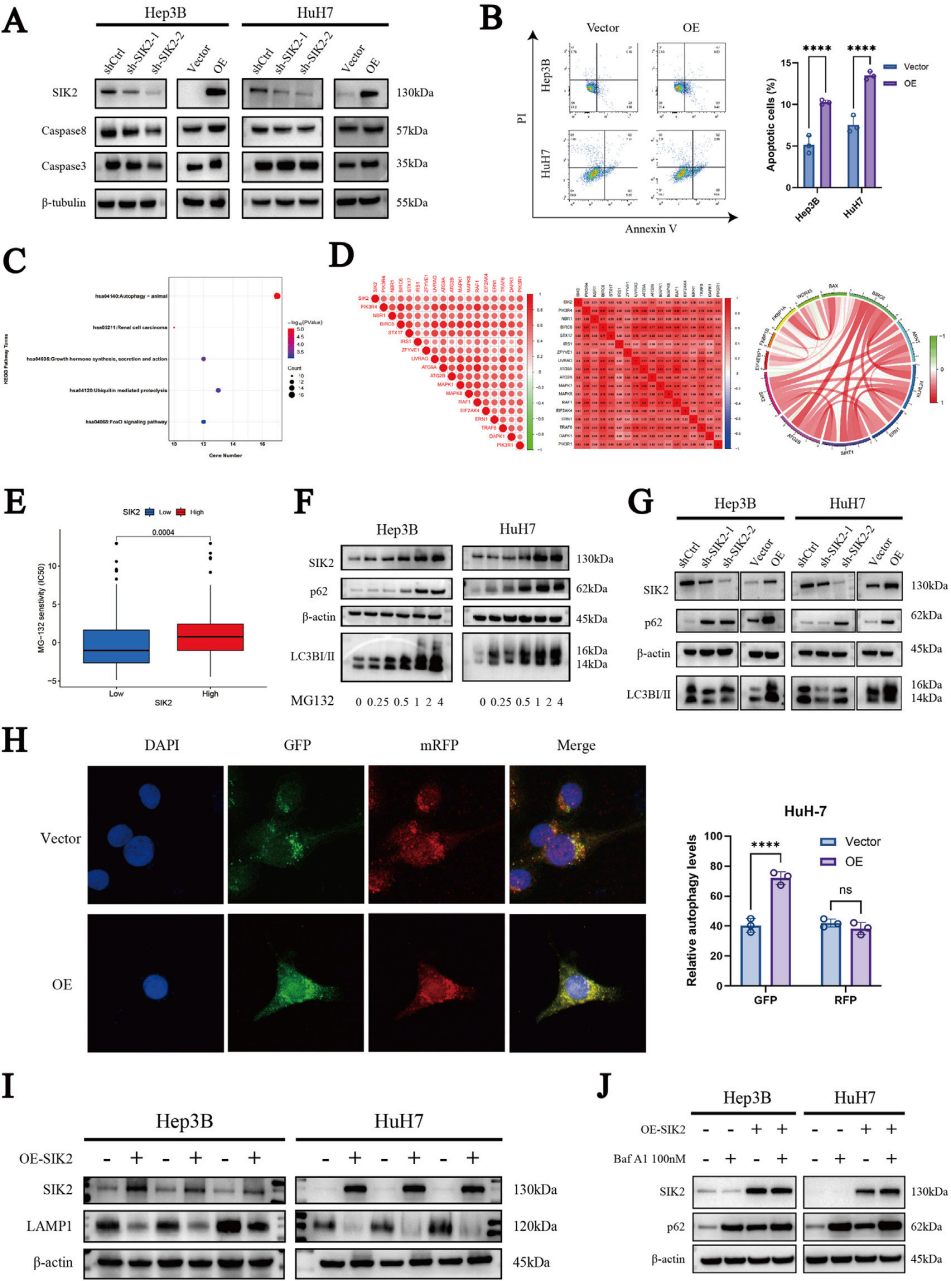
SIK2 is involved in regulating apoptosis and autophagy in HCC. **(A)** Western blot: effects of SIK2 knockdown/overexpression on caspase3, caspase8 (β-tubulin as control). **(B)** Flow cytometry analysis of the apoptosis proportions in Hep3B and HuH7 cell lines after SIK2 knockdown or overexpression. **(C)** KEGG enrichment of SIK2 co-expressed genes (correlation coefficient > 0.5, p < 0.05). **(D)** Co-expression of SIK2 and autophagy-related genes. **(E)** HCC cell sensitivity to MG132 by SIK2 expression. **(F)** Effects of MG132 concentration on SIK2 and autophagy proteins in Hep3B/HuH7 (β-actin as control). **(G)** Western blot: effects of SIK2 knockdown/overexpression on p62, LC3B (β-actin as control). **(H)** AdPlus-mCherry-GFP-LC3B assay: SIK2 overexpression on autophagy flux in HuH7 (quantified by ImageJ). **(I)** Western blot: SIK2, LAMP1 in Hep3B/HuH7 with SIK2 overexpression (β-actin as control). **(J)** Western blot: SIK2, p62 in Hep3B/HuH7 with SIK2 overexpression ± Bafilomycin A1 (100 nM). Data: mean ± SD/SEM (n = 3); analyzed by two-tailed unpaired t-test or ANOVA. *P < 0.05, **P < 0.01, ***P < 0.001, ****P < 0.0001.

To elucidate the mechanistic role of SIK2 in HCC cells, we first examined its regulatory effects on caspase-8 and caspase-3 via Western blotting. SIK2 overexpression significantly upregulated both caspase-8 and caspase-3 expression in HuH-7 and Hep3B cells compared with that in control cells. Conversely, SIK2 knockdown reduced caspase-8 levels without significantly affecting caspase-3 (Figure 4A), suggesting that SIK2 primarily modulates apoptosis through caspase-8 regulation, with caspase-3 activation potentially dependent on caspase-8 status. To further validate the impact of the SIK2 expression level on apoptosis in HCC, we analyzed the apoptosis rates of both cell lines via Annexin V/PI double-staining flow cytometry. These results, which are consistent with the Western blot findings, demonstrated that SIK2 overexpression significantly increased the apoptosis rate, confirming that SIK2 promotes apoptosis by activating the caspase-8/caspase-3 signaling pathway (Figure 4B).

Given the frequent interplay between apoptosis and autophagy, we further explored the role of SIK2 in autophagy-related pathways. First, we identified genes coexpressed with SIK2 (R > 0.5, *P* < 0.05) through bioinformatics analysis of TCGA-LIHC data. Subsequent KEGG pathway enrichment analysis revealed that these genes were predominantly associated with autophagy-related pathways (Figure 4C). Further correlation analysis revealed significant associations between SIK2 and core autophagy-related genes (Figure 4D).

Concurrently, our previous drug sensitivity analysis revealed that the high-SIK2-expressing group exhibited significantly greater sensitivity to MG132 than the low-SIK2-expressing group did, suggesting that elevated SIK2 enhances cellular susceptibility to MG132 (Figure 4E). To explore this further, we treated the Hep3B and HuH-7 cell lines with various concentrations of MG132 for 24 h and assessed the expression levels of SIK2, p62, and LC3B via Western blotting. The results revealed a dose-dependent increase in SIK2, p62, and LC3B expression as the MG132 concentration increased, indicating that SIK2 may participate in MG132-induced autophagy processes (Figure 4F).

To further validate the involvement of SIK2 in autophagy in HCC, we examined the effects of SIK2 knockdown or overexpression on p62 and LC3B protein expression in both cell lines via WB. In the SIK2-knockdown group, we observed reduced expression levels of LC3B-I and LC3B-II alongside elevated p62 expression. Conversely, in the SIK2 overexpression group, LC3B-I and LC3B-II expression increased, whereas p62 expression remained elevated (Figure 4G). These findings confirm the participation of SIK2 in HCC autophagy, although the abnormal upregulation of p62 warrants further investigation. To clarify the specific stages of autophagy influenced by SIK2, we employed the AdPlus-mCherry-GFP-LC3B autophagic flux assay. The results aligned with the WB data: in the SIK2 overexpression group, increased GFP signal intensity indicated improved autophagosome formation efficiency (e.g., LC3-II-positive structures). However, the intensity of the RFP signal (lysosomal marker) did not significantly increase, suggesting that autophagosome‒lysosome fusion efficiency was not proportionally increased and might even be suppressed despite increased numbers of autophagosomes (Figure 4H). In response to this anomaly, we evaluated lysosomal function. Western blotting results indicated that SIK2 overexpression significantly inhibited Lysosome-associated membrane protein 1 (LAMP1) (Figure 4I). We further validated our hypothesis using the autophagy inhibitor Bafilomycin A1 (Baf A1). The results showed that the accumulation of p62 was significantly reduced in the SIK2 overexpression group compared to the control group (Figure 4J), indicating that the autophagic flux was blocked in the context of SIK2 overexpression. In summary, SIK2 plays a dual regulatory role in HCC autophagy. It positively regulates autophagosome formation while concurrently inhibiting lysosomal degradation at later stages.

### 3.5 Experimental validation of SP1 as an upstream transcriptional regulator of SIK2

**FIGURE 5:**
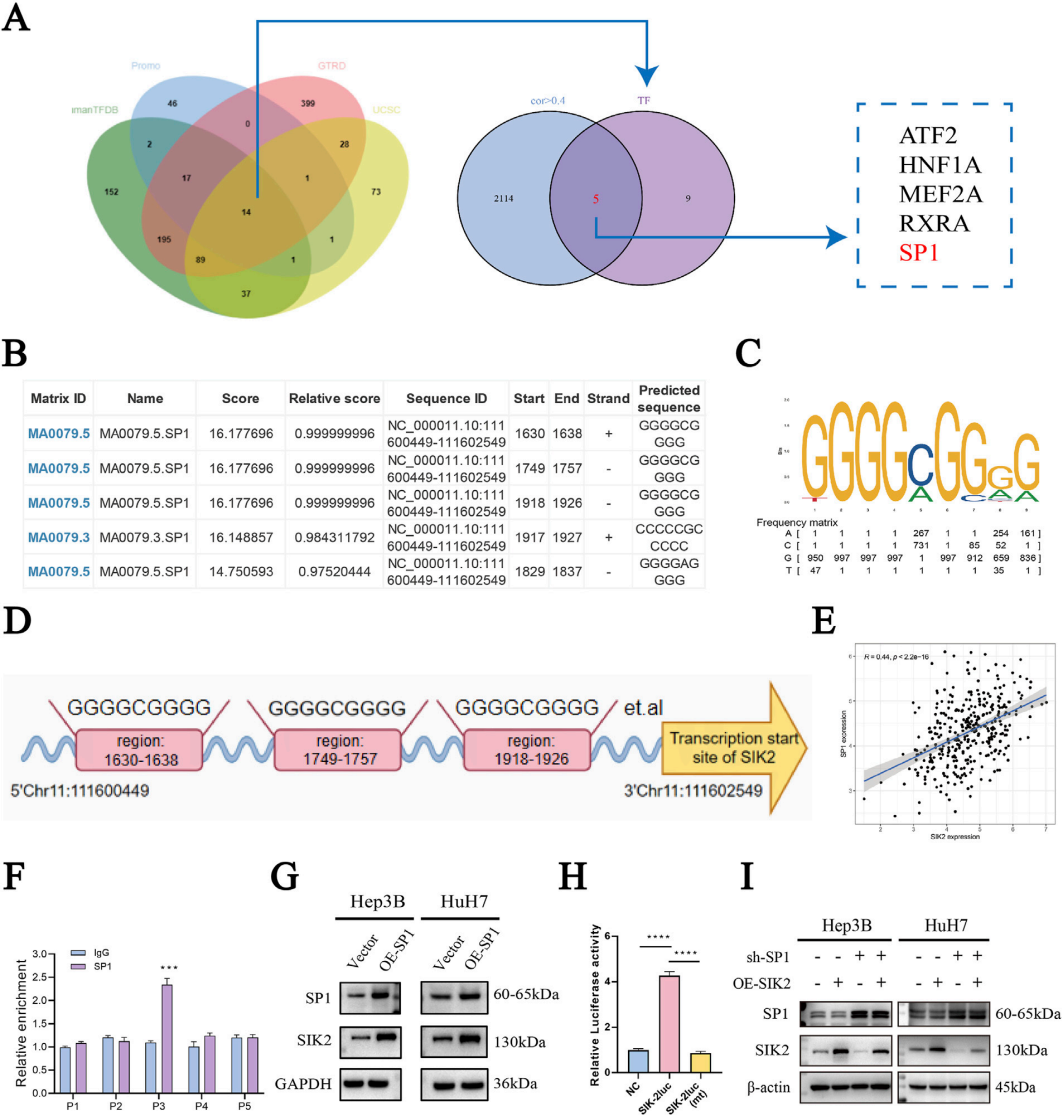
SIK2 is under direct regulation by the transcription factor SP1 in HCC. **(A)** Bioinformatics: SP1 as a potential SIK2 transcription factor. **(B)** Top 6 SIK2 promoter binding sites predicted by JASPAR (based on relative scores). **(C,D)** Putative SP1-binding sites in SIK2 promoter region. **(E)** TCGA Pearson correlation: positive correlation between SIK2 and SP1 mRNA. **(F)** ChIP-qRT-PCR: SP1 binds to SIK2 promoter. **(G)** SIK2 protein reduced with SP1 overexpression. **(H)** Dual-luciferase assay: SP1-binding site mutation abolishes SP1 action on SIK2 promoter. **(I)** Western blot: SP1, SIK2 in Hep3B/HuH7 with SP1 knockdown (sh-SP1) ± SIK2 overexpression (OE-SIK2). Data: mean ± SD/SEM (n = 3); analyzed by two-tailed unpaired t-test or ANOVA. *P < 0.05, **P < 0.01, ***P < 0.001, ****P < 0.0001.

To systematically identify upstream transcriptional regulators of SIK2 and elucidate its molecular regulatory mechanisms, we integrated prediction results from the HumanTFDB, GTRD, and PROMO databases, along with UCSC platform-based analyses combined with JASPAR database screening. This approach led to the screening of five transcription factors (ATF2, HNF1A, MEF2A, RXRA, and SP1) that presented correlation coefficients greater than 0.4 with SIK2 and were consistently predicted across multiple databases (Figure 5A). Further refinement via the JASPAR database prioritized SP1, as DNA-binding site predictions revealed multiple high-affinity SP1-binding motifs within the SIK2 promoter region. The predicted binding sites included NC_000011.10:1,630–1,638, 1749–1757, 1918–1926, 1917–1927, and 1829–1837, with relative scores approaching 1, indicating strong binding potential (Figure 5B). Sequence analysis of these sites revealed the canonical SP1-binding motif “GGGCGGGG,” characterized by a high frequency of G/C nucleotides, which is consistent with the known binding preferences of SP1 (Figures 5C,D). Correlation analysis of the TCGA-LIHC data revealed a significant positive association between the SP1 and SIK2 expression levels (R = 0.44, *P* < 0.0001) (Figure 5E). These results suggest that SP1 is closely related to the regulation of the transcriptional activity and expression levels of SIK2. Chromatin immunoprecipitation (ChIP) coupled with qRT‒PCR confirmed enriched SP1 binding at the predicted SIK2 promoter regions compared with nonspecific IgG controls (Figure 5F). Western blot analysis further confirmed that SP1 overexpression markedly increased SIK2 protein levels (Figure 5G). Dual-luciferase reporter assays revealed that SP1 overexpression significantly enhanced SIK2 promoter-driven luciferase activity (Figure 5H). Rescue experiments revealed that SIK2 overexpression did not affect SP1 levels. Conversely, knockdown of SP1 in SIK2-overexpressing cells resulted in decreased SIK2 expression compared to the SIK2 overexpression control group (Figure 5I). Collectively, these findings demonstrate that SP1 directly binds to the SIK2 promoter, regulates its transcriptional activity, and modulates SIK2 expression levels, establishing that SP1 is a critical upstream transcriptional regulator of SIK2 in HCC.

**FIGURE 6:**
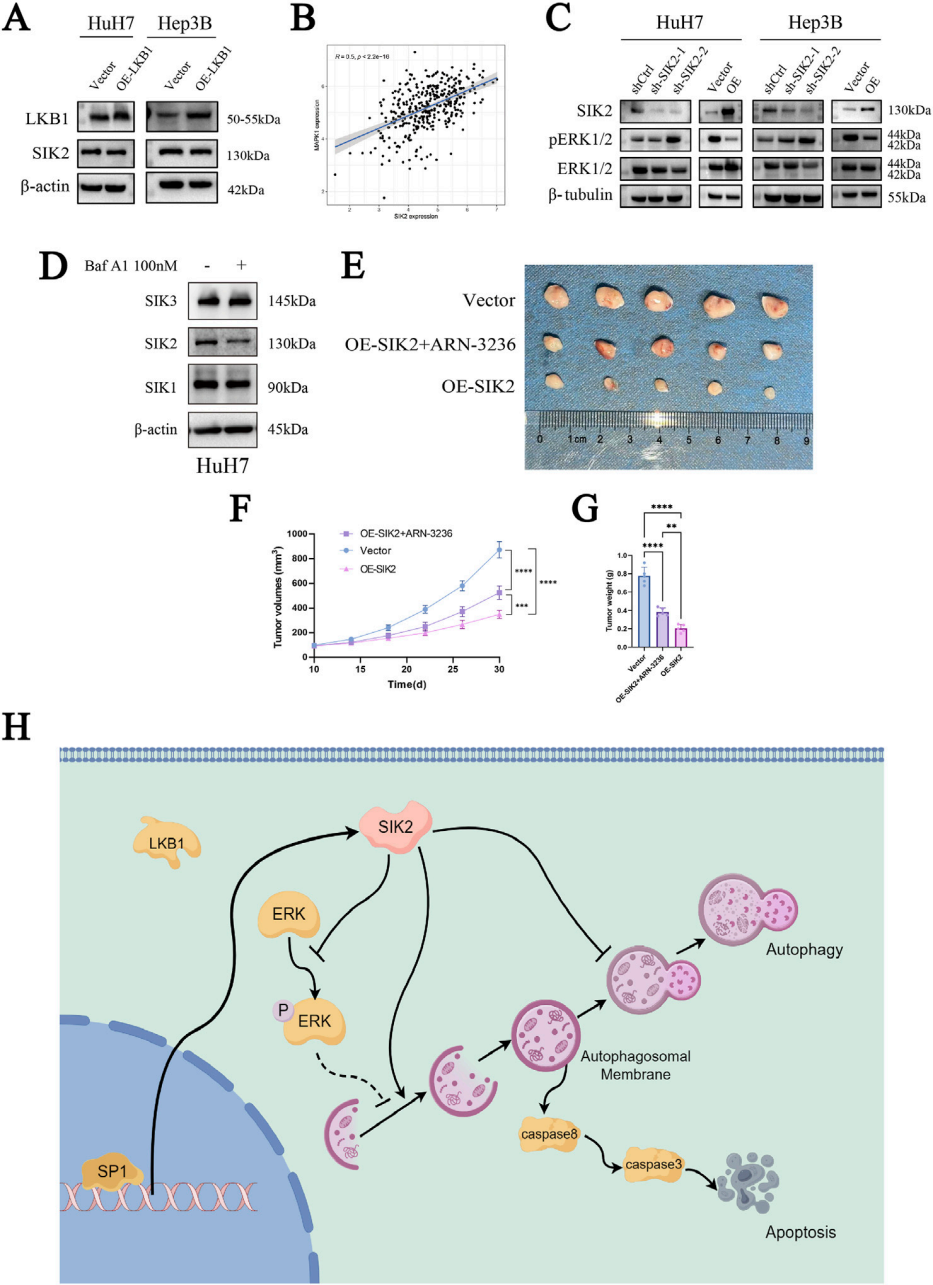
SIK2 may serve as a potential biomarker for clinical intervention in HCC. **(A)** SIK2 protein unchanged with LKB1 overexpression (β-actin as control). **(B)** TCGA Pearson correlation: positive correlation between SIK2 and MAPK1 mRNA. **(C)** Western blot: effects of SIK2 knockdown/overexpression on ERK1/2, pERK1/2. **(D)** Western blot: SIK family members with/without Baf A1. **(E)** Representative xenograft tumor images: nude mice with HuH-7-transfected cells (n = 5). **(F)** Xenograft tumor volume curves (n = 5). **(G)** Xenograft tumor weight statistics (n = 5). **(H)** Regulated by the transcription factor SP1, SIK2 initiates autophagic responses by activating the AMPK/mTORC1 pathway and suppressing ERK signaling to enhance autophagosome formation. However, impaired autophagic flux—manifested by p62 accumulation due to lysosomal dysfunction—triggers apoptosis through caspase-8/-3 activation. Data: mean ± SD/SEM (n = 3); analyzed by two-tailed unpaired t-test or ANOVA. *P < 0.05, **P < 0.01, ***P < 0.001, ****P < 0.0001.

Moreover, in our investigation of SIK2, we noted that LKB1 regulates the phosphorylation of SIK2. To investigate whether SIK2 expression levels are influenced by LKB1 and thereby involved in autophagy, we overexpressed LKB1 in the HuH-7 and Hep3B cell lines. Western blot analysis of LKB1 and SIK2 expression levels revealed that LKB1 overexpression did not significantly alter SIK2 protein levels (Figure 6A).

Our previous KEGG enrichment analysis revealed that SIK2 was enriched in the MAPK/ERK-associated signaling pathway. To determine whether SIK2 modulates autophagy through the MAPK/ERK pathway, we performed a correlation analysis between SIK2 and ERK (MAPK1). As anticipated, the SIK2 expression level was significantly positively correlated with the ERK expression level (R ≈ 0.5, *P* < 0.0001) (Figure 6B). We subsequently examined the protein expression levels of ERK1/2 and phosphorylated ERK1/2 (pERK1/2) in various cell lines following SIK2 knockdown or overexpression. We found that SIK2 overexpression reduced both ERK1/2 and pERK1/2 protein levels. Conversely, SIK2 knockdown increased pERK1/2 levels, whereas ERK1/2 expression did not significantly change (Figure 6C). These findings suggest that SIK2 may influence downstream signaling pathways by suppressing ERK phosphorylation, thereby modulating autophagy in HCC.

### 3.6 SIK2 may serve as a potential biomarker for clinical intervention in HCC

Finally, to preliminarily validate the clinical translational significance of SIK2 as a potential diagnostic and therapeutic target for HCC, we employed ARN-3236, a specific small-molecule inhibitor of SIK2, and performed subcutaneous xenograft tumor experiments in nude mice in the control (Vector) and SIK2-overexpressing (OE-SIK2) groups. Additionally, Western blotting was performed to validate the specificity of ARN-3236 as a SIK2 inhibitor (Figure 6D). Consistent with previous *in vitro* findings, mice injected with control HuH-7 cells (vector) developed tumors with significantly greater weights than those injected with HuH-7 cells stably overexpressing SIK2 (OE-SIK2). The tumor volume curves revealed that, compared with those in the OE-SIK2 group, the tumors in the vector group increased markedly over time and grew at a significantly faster rate. Intriguingly, when ARN-3236 was coadministered to the OE-SIK2 group, the tumorigenic capacity of the mice was significantly restored (Figures 6E–G). These results collectively indicate that SIK2 overexpression similarly exerts tumor-suppressive effects in *in vivo* HCC models.

## 4 Discussion

SIK2, a serine/threonine kinase belonging to the AMPK family, is widely expressed in metabolically active organs, including the brain, liver, adipose tissue, and skeletal muscle (Feldman et al., 2000). Its expression is dynamically modulated by metabolic signals such as salt, insulin, and glucose (Horike et al., 2003; Park et al., 2014; Darling and Cohen, 2021). Functionally, SIK2 serves as a critical regulator of cellular energy homeostasis through orchestrating key biological processes ranging from autophagy and apoptosis to systemic metabolic adaptation (Wang et al., 2016; Sun et al., 2020). SIK2 has been demonstrated to play dual roles across different tumor types, acting as an oncogenic driver that promotes tumor progression in certain malignancies while functioning as a tumor suppressor that inhibits neoplastic growth in others (Zhao et al., 2020; Lu et al., 2022; Rong et al., 2022; Shi et al., 2022; Yu et al., 2023). In a study by GAO *et al.*, SIK2 was shown to play a critical oncogenic role in promoting ovarian cancer growth and metastasis through activation of the PI3K/AKT/HIF-1α signaling pathway and induction of mitochondrial fission (Gao et al., 2020). In contrast, BON *et al.* reported that SIK2 regulates mitotic progression and transcription in preadenocarcinoma, inhibits cancer cell growth, and induces cell cycle arrest and apoptosis (Bon et al., 2015). Previous studies on liver diseases have extensively investigated SIK2 in the context of glucose and fatty acid metabolism, insulin resistance, and chronic inflammation (Bricambert et al., 2010; Li et al., 2021). However, the specific mechanisms underlying its role in the initiation and progression of HCC remain systematically underexplored. In recent years, the rapid advancement of bioinformatics tools and databases has emerged as a critical asset in disease genomics research, offering novel perspectives and theoretical foundations to advance studies on HCC(Roos, 2001). This study conducted a series of bioinformatics analyses on SIK2, including survival analysis, clinical correlation analysis, independent prognostic analysis, tumor microenvironment analysis, immune cell infiltration, and immunotherapy analysis. The results revealed that SIK2, an independent prognostic factor closely associated with patient outcomes, plays a significant cancer-suppressive role in HCC. In-depth exploration of the tumor microenvironment and immune infiltration demonstrated that high SIK2 expression may exert antitumor effects by remodeling the tumor immune microenvironment. The significantly elevated immune scores and positive correlations with antitumor immune cells, coupled with the negative regulatory characteristics of immunosuppressive M2 macrophages, suggest that SIK2 may enhance antitumor immune responses by promoting the infiltration of immunologically active cells while suppressing the formation of an immunosuppressive microenvironment. These findings provide new perspectives for understanding the molecular mechanisms of SIK2 in tumor immunoregulation and indicate that targeting SIK2 could become a potential strategy for improving the tumor immune microenvironment (Clark et al., 2012; Darling et al., 2017; Zhu et al., 2023). To investigate the functional significance of SIK2 in HCC cells, we systematically analyzed its regulatory effects on cell proliferation, migration, invasion, and apoptosis by constructing SIK2-overexpressing and SIK2-knockdown cell models, confirming its tumor-suppressive characteristics in HCC. Notably, SIK2 knockdown reduced caspase-8 expression without significantly altering caspase-3 levels, while SIK2 overexpression upregulated both caspases. We propose that this observation reflects a hierarchical organization of apoptotic signaling in HCC cells. SIK2’s regulation of apoptosis exhibits a layered structure: caspase-8 is the primary regulatory node, functioning as the initiator caspase within the extrinsic apoptotic pathway, whereas caspase-3 (an effector caspase) activation depends on upstream caspase-8 expression. Consequently, SIK2 can stabilize caspase-8 while indirectly influencing caspase-3. Although this study did not examine other regulatory factors, such as Bcl-2 family proteins, this hierarchical profile underscores the redundancy and compensatory mechanisms of the apoptotic signaling cascade in tumor cells. It suggests that SIK2 may induce apoptosis through the extrinsic apoptotic pathway, consistent with prior research (Ashkenazi, 2015). These findings lay the foundation for subsequent research to elucidate the role of SIK2 in the autophagy‒apoptosis regulatory network.

Autophagy is a highly conserved self-degradation mechanism within cells that primarily maintains cellular homeostasis and functionality by degrading damaged organelles, proteins, and other macromolecules. The autophagic process is divided into distinct phases: the initial phase, the nucleation phase, the elongation and closure phase, the maturation phase, and the degradation and recycling phase (Thoen et al., 2011; Mizushima and Levine, 2020). This dynamic process involves the regulation of more than 40 autophagy-related genes (Levine and Kroemer, 2019; Zhao and Zhang, 2019). Notably, AMPK acts as a key molecular switch during the initiation phase by sensing intracellular ATP levels to regulate ULK1 phosphorylation, thereby triggering autophagy activation (Kim et al., 2011). As a member of the AMPK family, the regulatory role of SIK2 in autophagy has been well documented (Yang et al., 2013; Xu et al., 2022; Zhang et al., 2022). However, the mechanistic underpinnings of this regulation exhibit significant heterogeneity across distinct cell types and pathological contexts. For example, Maxfield *et al.* demonstrated that SIK2 restricts autophagic flux to support the survival of triple-negative breast cancer (TNBC) cells, particularly the claudin-low subtype, and that its inhibition reactivates autophagy, inducing selective tumor cell death (Maxfield et al., 2016). In contrast, DAI *et al.* reported that SIK2 inhibits AKT/GSK3β/β-catenin signaling by restraining the autophagic degradation of protein phosphatases, ultimately suppressing gastric cancer (Dai et al., 2021). These findings suggest that SIK2 may exert differential regulatory effects by targeting distinct autophagy phases (e.g., initiation vs lysosomal degradation), although its functional role in HCC remains undefined. Therefore, we analyzed the regulatory role of SIK2 in autophagy in HCC cells. Through analysis of the TCGA-LIHC database, we found that SIK2 coexpressed genes (*R* > 0.5) were significantly enriched in autophagy-related pathways and strongly correlated with core autophagy-related genes. Drug sensitivity analysis revealed that HCC cells with high SIK2 expression were more sensitive to the proteasome inhibitor MG132, a commonly used autophagy activator that blocks the ubiquitin–proteasome system (UPS) from accumulating misfolded proteins, thereby triggering compensatory autophagy for clearance (Yuan et al., 2008; Harhouri et al., 2017; Shu et al., 2024). Combined with the WB results, the dose-dependent accumulation of SIK2, LC3B-II, and p62 after MG132 treatment indicated that SIK2 participates in the adaptive regulation of cellular responses to autophagic stress. Mechanistic studies revealed that SIK2 knockdown led to reduced LC3B-I/LC3B-II expression and increased p62 accumulation, suggesting impaired autophagosome formation and blocked autophagic degradation. Conversely, SIK2 overexpression significantly elevated LC3B-I/LC3B-II levels, increased p62 expression, and inhibited LAMP1 expression. This unique autophagosome accumulation phenomenon suggests a potential link to apoptotic outcomes, revealing a “dual regulatory” role of SIK2 in autophagic flux: 1. Initiation phase: SIK2 enhances autophagosome formation by activating upstream signaling pathways (e.g., the AMPK/mTOR/ULK1 axis). 2. Degradation phase: Potential lysosomal dysfunction (as SIK2 overexpression significantly inhibited LAMP1 expression in our experiments, suggesting reduced autophagosome-lysosome fusion efficiency and impaired lysosomal degradative capacity) hindered late-stage autophagic flux, ultimately leading to aberrant p62 accumulation (Xu and Ren, 2015; Ballabio and Bonifacino, 2020; Yang et al., 2020). Baf A1, a macrolide antibiotic, is employed in cellular autophagy studies to impede the fusion of autophagosomes and lysosomes, consequently hindering autophagosome degradation. To cross-validate the mechanism of SIK2, we utilized Baf A1 treatment. Our findings revealed that autophagic flux was obstructed under conditions of SIK2 overexpression. This saturation of the degradation step meant that the inhibitor failed to induce additional p62 accumulation, thereby providing more robust support for our conjecture. In previous studies, autophagy and apoptosis exhibited complex interaction patterns, which can be either antagonistic or synergistic (Shimizu et al., 2004; Yu et al., 2004; Maiuri et al., 2007; Sukumaran et al., 2021; Biswas et al., 2024). Young *et al.* revealed that autophagosome membranes serve as platforms for intracellular death-inducing signaling complexes (iDISC), which recruit caspase-8 to initiate caspase-8/-3 cascades, resulting in autophagy-dependent apoptosis (Young et al., 2012). Combined with our research results, the blockade of autophagy flux-induced autophagosome accumulation may ultimately trigger apoptosis through the interaction between autophagosome membranes and caspase-8, which may be the molecular basis for the SIK2-mediated inhibition of tumor growth. This discovery not only expands the spatiotemporal-specific understanding of SIK2 within the autophagy regulatory network but also provides novel perspectives for precisely targeted interventions at distinct stages of autophagy flux.

Furthermore, to systematically dissect the functional regulatory network of SIK2 in HCC, we analyzed its potentially involved transcriptional regulatory mechanisms and the activity profiles of associated signaling pathways. ERK (extracellular signal-regulated kinase), a core component of the MAPK signaling pathway, transmits growth factor and stress signals through phosphorylation cascades to regulate cellular proliferation, differentiation, and autophagy. Its activation status is typically characterized by phosphorylated ERK (pERK) levels (Fang and Richardson, 2005; Moon and Ro, 2021; Ullah et al., 2022). Our study revealed that SIK2 overexpression significantly reduced pERK (phosphorylated ERK) levels, whereas SIK2 knockdown led to abnormal pERK accumulation, suggesting that SIK2 regulates ERK phosphorylation. On the basis of these theoretical and experimental findings, we propose that SIK2 coordinately regulates autophagy in HCC cells through two distinct mechanisms: 1. AMPK pathway-dependent mechanism: The increased LC3B-II conversion observed in SIK2-overexpressing cells indicates that SIK2 may promote autophagosome formation via canonical AMPK signaling pathways, such as mTORC1 inhibition or direct ULK1 phosphorylation. 2. The negative regulatory mechanism of ERK signaling is as follows: SIK2 suppresses ERK phosphorylation, thereby alleviating its inhibitory effects on autophagy and establishing a synergistic regulatory network (Cagnol and Chambard, 2010; Wang et al., 2019; Huang et al., 2023). With respect to the upstream regulatory mechanism of SIK2, LKB1, a classical activator of the AMPK family, can suppress tumorigenesis by coordinating the growth–metabolism balance. However, our study revealed that overexpression of LKB1 in HCC cell lines did not significantly alter SIK2 protein levels, suggesting the existence of non-LKB1-dependent regulatory pathways (Alessi et al., 2006; Nguyen et al., 2023). To elucidate the mechanistic basis of SIK2 transcriptional regulation, we integrated multidatabase predictions with JASPAR bioinformatics analyses, identifying SP1 as a transcription factor with high-affinity binding sites in the SIK2 promoter region and revealing a significant positive correlation between the expression levels of these genes. ChIP‒qPCR confirmed the specific binding of SP1 to the SIK2 promoter (compared with the IgG control), while Western blotting further validated the transcriptional regulatory role of SP1 over SIK2. Notably, SP1, a widely expressed zinc-finger transcription factor, has displayed a dual role in previous HCC research, acting as both an oncogene and a tumor suppressor (Vizcaíno et al., 2015; Su et al., 2024; Wang et al., 2024), a characteristic that highlights its significant background dependency and specificity. By modulating numerous downstream molecules like EPS8L3 and SQLE, SP1 impacts HCC progression (Tsui et al., 2024; Xiao et al., 2025). Our research provides new insights into SP1’s complex functions by demonstrating its tumor-suppressive activity through the activation of SIK2 transcription.

Finally, in a nude mouse xenograft model, we successfully validated that SIK2 overexpression can inhibit liver cancer *in vivo*. The specificity of the SIK2 inhibitor ARN-3236 (Lombardi et al., 2016; Zhou et al., 2017) toward SIK2 was validated in a murine setting. Injection of ARN-3236 effectively suppressed SIK2 and promoted tumor growth. Our animal studies underscore the potential clinical translational value of SIK2 as a diagnostic and therapeutic target for hepatocellular carcinoma. Nevertheless, it is undeniable that SIK2 has a wide range of biological functions, and a more comprehensive research evaluation is still needed to assess potential risks in the more complex human environment.

In summary, this study systematically elucidates the tumor-suppressive role of SIK2 in HCC and its molecular mechanisms, encompassing regulation of the immune microenvironment, induction of apoptosis-autophagic flux imbalance, transcriptional control, and signaling pathway crosstalk. From a translational medicine perspective, SIK2 activators or their combination with immune checkpoint inhibitors may emerge as novel therapeutic strategies for HCC. Future investigations should focus on delineating SIK2’s precise mechanisms in immune evasion and advancing its clinical translational research to provide new directions for precision therapy in liver cancer.

## Data Availability

The raw data supporting the conclusions of this article will be made available by the authors, without undue reservation.

## Abbreviations

HCC, hepatocellular carcinoma; SIK2, Salt-inducible kinase 2; AMPK, AMP-activated protein kinase; IHC, immunohistochemistry; LKB1, liver kinase B1; TCGA, The Cancer Genome Atlas; TME, tumor microenvironment; KEGG, Kyoto Encyclopedia of Genes and Genomes; ATCC, the American Type Culture Collection; DMEM, Dulbecco’s modified Eagle’s medium; FBS, fetal bovine serum; qRT‒PCR, Quantitative real-time PCR; WB, Western blotting; ECL, enhanced chemiluminescence; CCK-8, Cell Counting Kit-8; PFA, Paraformaldehyde; ChIP‒qPCR, Chromatin immunoprecipitation-quantitative polymerase chain reaction; HPA, the human protein atlas; OS, overall survival; HR, hazard ratio; TCIA, The Cancer Imaging Archive; RFP, red fluorescent protein; GFP, green fluorescent protein; UPS, ubiquitin–proteasome system; iDISC, intracellular death-inducing signaling complexes; ERK, extracellular signal-regulated kinase.

## References

[B1] Alessi D. R. Sakamoto K. Bayascas J. R. (2006). LKB1-Dependent signaling pathways. Annu. Rev. Biochem. 75, 137–163. 10.1146/annurev.biochem.75.103004.142702 16756488

[B2] Amir M. Zhao E. Fontana L. Rosenberg H. Tanaka K. Gao G. (2013). Inhibition of hepatocyte autophagy increases tumor necrosis factor-dependent liver injury by promoting caspase-8 activation. Cell Death Differ. 20, 878–887. 10.1038/cdd.2013.21 23519075 PMC3679456

[B3] Ashkenazi A. (2015). Targeting the extrinsic apoptotic pathway in cancer: lessons learned and future directions. J. Clin. Invest 125, 487–489. 10.1172/JCI80420 25642709 PMC4319431

[B4] Ballabio A. Bonifacino J. S. (2020). Lysosomes as dynamic regulators of cell and organismal homeostasis. Nat. Rev. Mol. Cell Biol. 21, 101–118. 10.1038/s41580-019-0185-4 31768005

[B5] Biswas U. Roy R. Ghosh S. Chakrabarti G. (2024). The interplay between autophagy and apoptosis: its implication in lung cancer and therapeutics. Cancer Lett. 585, 216662. 10.1016/j.canlet.2024.216662 38309614

[B6] Bon H. Wadhwa K. Schreiner A. Osborne M. Carroll T. Ramos-Montoya A. (2015). Salt-inducible kinase 2 regulates mitotic progression and transcription in prostate cancer. Mol. Cancer Res. 13, 620–635. 10.1158/1541-7786.MCR-13-0182-T 25548099 PMC4383640

[B7] Bray F. Laversanne M. Sung H. Ferlay J. Siegel R. L. Soerjomataram I. (2024). Global cancer statistics 2022: GLOBOCAN estimates of incidence and mortality worldwide for 36 cancers in 185 countries. CA Cancer J. Clin. 74, 229–263. 10.3322/caac.21834 38572751

[B8] Bricambert J. Miranda J. Benhamed F. Girard J. Postic C. Dentin R. (2010). Salt-inducible kinase 2 links transcriptional coactivator p300 phosphorylation to the prevention of ChREBP-dependent hepatic steatosis in mice. J. Clin. Invest 120, 4316–4331. 10.1172/JCI41624 21084751 PMC2993582

[B9] Cagnol S. Chambard J.-C. (2010). ERK and cell death: mechanisms of ERK-Induced cell death--apoptosis, autophagy and senescence. FEBS J. 277, 2–21. 10.1111/j.1742-4658.2009.07366.x 19843174

[B10] Clark K. MacKenzie K. F. Petkevicius K. Kristariyanto Y. Zhang J. Choi H. G. (2012). Phosphorylation of CRTC3 by the salt-inducible kinases controls the interconversion of classically activated and regulatory macrophages. Proc. Natl. Acad. Sci. U. S. A. 109, 16986–16991. 10.1073/pnas.1215450109 23033494 PMC3479463

[B11] Dai X. Zhang Y. Lin X. Huang X. Zhang Y. Xue C. (2021). SIK2 represses AKT/GSK3β/β‐catenin signaling and suppresses gastric cancer by inhibiting autophagic degradation of protein phosphatases. Mol. Oncol. 15, 228–245. 10.1002/1878-0261.12838 33128264 PMC7782074

[B12] Darling N. J. Cohen P. (2021). Nuts and bolts of the salt-inducible kinases (SIKs). Biochem. J. 478, 1377–1397. 10.1042/BCJ20200502 33861845 PMC8057676

[B13] Darling N. J. Toth R. Arthur J. S. C. Clark K. (2017). Inhibition of SIK2 and SIK3 during differentiation enhances the anti-inflammatory phenotype of macrophages. Biochem. J. 474, 521–537. 10.1042/BCJ20160646 27920213 PMC5290485

[B14] Debnath J. Gammoh N. Ryan K. M. (2023). Autophagy and autophagy-related pathways in cancer. Nat. Rev. Mol. Cell Biol. 24, 560–575. 10.1038/s41580-023-00585-z 36864290 PMC9980873

[B15] Du W.-Q. Zheng J.-N. Pei D.-S. (2016a). The diverse oncogenic and tumor suppressor roles of salt-inducible kinase (SIK) in cancer. Expert Opin. Ther. Targets 20, 477–485. 10.1517/14728222.2016.111452 26549013

[B16] Du W.-Q. Zheng J.-N. Pei D.-S. (2016b). The diverse oncogenic and tumor suppressor roles of salt-inducible kinase (SIK) in cancer. Expert Opin. Ther. Targets 20, 477–485. 10.1517/14728222.2016.1101452 26549013

[B17] Fang J. Y. Richardson B. C. (2005). The MAPK signalling pathways and colorectal cancer. Lancet Oncol. 6, 322–327. 10.1016/S1470-2045(05)70168-6 15863380

[B18] Feldman J. D. Vician L. Crispino M. Hoe W. Baudry M. Herschman H. R. (2000). The salt-inducible kinase, SIK, is induced by depolarization in brain. J. Neurochem. 74, 2227–2238. 10.1046/j.1471-4159.2000.0742227.x 10820182

[B19] Gao T. Zhang X. Zhao J. Zhou F. Wang Y. Zhao Z. (2020). SIK2 promotes reprogramming of glucose metabolism through PI3K/AKT/HIF-1α pathway and Drp1-mediated mitochondrial fission in ovarian cancer. Cancer Lett. 469, 89–101. 10.1016/j.canlet.2019.10.029 31639424

[B20] González-Polo R.-A. Boya P. Pauleau A.-L. Jalil A. Larochette N. Souquère S. (2005). The apoptosis/autophagy paradox: autophagic vacuolization before apoptotic death. J. Cell Sci. 118, 3091–3102. 10.1242/jcs.02447 15985464

[B21] Han Z. Liu D. Chen L. He Y. Tian X. Qi L. (2021). PNO1 regulates autophagy and apoptosis of hepatocellular carcinoma via the MAPK signaling pathway. Cell Death Dis. 12, 552. 10.1038/s41419-021-03837-y 34050137 PMC8163843

[B22] Harhouri K. Navarro C. Depetris D. Mattei M.-G. Nissan X. Cau P. (2017). MG132-induced progerin clearance is mediated by autophagy activation and splicing regulation. EMBO Mol. Med. 9, 1294–1313. 10.15252/emmm.201607315 28674081 PMC5582415

[B23] Horike N. Takemori H. Katoh Y. Doi J. Min L. Asano T. (2003). Adipose-specific expression, phosphorylation of Ser794 in insulin receptor substrate-1, and activation in diabetic animals of salt-inducible kinase-2. J. Biol. Chem. 278, 18440–18447. 10.1074/jbc.M211770200 12624099

[B24] Huang Y. Zhen Y. Chen Y. Sui S. Zhang L. (2023). Unraveling the interplay between RAS/RAF/MEK/ERK signaling pathway and autophagy in cancer: from molecular mechanisms to targeted therapy. Biochem. Pharmacol. 217, 115842. 10.1016/j.bcp.2023.115842 37802240

[B25] Kim J. Kundu M. Viollet B. Guan K.-L. (2011). AMPK and mTOR regulate autophagy through direct phosphorylation of Ulk1. Nat. Cell Biol. 13, 132–141. 10.1038/ncb2152 21258367 PMC3987946

[B26] Koren E. Fuchs Y. (2021). Modes of regulated cell death in cancer. Cancer Discov. 11, 245–265. 10.1158/2159-8290.CD-20-0789 33462123

[B27] Levine B. Kroemer G. (2019). Biological functions of autophagy genes: a disease perspective. Cell 176, 11–42. 10.1016/j.cell.2018.09.048 30633901 PMC6347410

[B28] Li X. He S. Ma B. (2020). Autophagy and autophagy-related proteins in cancer. Mol. Cancer 19, 12. 10.1186/s12943-020-1138-4 31969156 PMC6975070

[B29] Li Y. Yu J. Jia M. Ma P. Dong C. (2021). Salt‐inducible kinase 2 functions as a tumor suppressor in hepatocellular carcinoma. Environ. Toxicol. 36, 2530–2540. 10.1002/tox.23366 34491613

[B30] Lizcano J. M. Göransson O. Toth R. Deak M. Morrice N. A. Boudeau J. (2004). LKB1 is a master kinase that activates 13 kinases of the AMPK subfamily, including MARK/PAR-1. EMBO J. 23, 833–843. 10.1038/sj.emboj.7600110 14976552 PMC381014

[B31] Llovet J. M. Kelley R. K. Villanueva A. Singal A. G. Pikarsky E. Roayaie S. (2021). Hepatocellular carcinoma. Nat. Rev. Dis. Prim. 7, 6. 10.1038/s41572-020-00240-3 33479224 PMC13537433

[B32] Lombardi M. S. Gilliéron C. Dietrich D. Gabay C. (2016). SIK inhibition in human myeloid cells modulates TLR and IL-1R signaling and induces an anti-inflammatory phenotype. J. Leukoc. Biol. 99, 711–721. 10.1189/jlb.2A0715-307R 26590148

[B33] Lu Z. Mao W. Yang H. Santiago-O’Farrill J. M. Rask P. J. Mondal J. (2022). SIK2 inhibition enhances PARP inhibitor activity synergistically in ovarian and triple-negative breast cancers. J. Clin. Investigation 132, e146471. 10.1172/JCI146471 35642638 PMC9151707

[B34] Maiuri M. C. Zalckvar E. Kimchi A. Kroemer G. (2007). Self-eating and self-killing: crosstalk between autophagy and apoptosis. Nat. Rev. Mol. Cell Biol. 8, 741–752. 10.1038/nrm2239 17717517

[B35] Mariño G. Niso-Santano M. Baehrecke E. H. Kroemer G. (2014). Self-consumption: the interplay of autophagy and apoptosis. Nat. Rev. Mol. Cell Biol. 15, 81–94. 10.1038/nrm3735 24401948 PMC3970201

[B36] Maxfield K. E. Macion J. Vankayalapati H. Whitehurst A. W. (2016). SIK2 restricts autophagic flux to support triple-negative breast cancer survival. Mol. Cell. Biol. 36, 3048–3057. 10.1128/MCB.00380-16 27697861 PMC5126292

[B37] Mizushima N. Komatsu M. (2011). Autophagy: renovation of cells and tissues. Cell 147, 728–741. 10.1016/j.cell.2011.10.026 22078875

[B38] Mizushima N. Levine B. (2020). Autophagy in human diseases. N. Engl. J. Med. 383, 1564–1576. 10.1056/NEJMra2022774 33053285

[B39] Mohammed W. H. Sulaiman G. M. Abomughaid M. M. Klionsky D. J. Abu-Alghayth M. H. (2024). The dual role of autophagy in suppressing and promoting hepatocellular carcinoma. Front. Cell Dev. Biol. 12, 1472574. 10.3389/fcell.2024.1472574 39463763 PMC11502961

[B40] Moon H. Ro S. W. (2021). MAPK/ERK signaling pathway in hepatocellular carcinoma. Cancers 13, 3026. 10.3390/cancers13123026 34204242 PMC8234271

[B41] Nguyen K. Hebert K. McConnell E. Cullen N. Cheng T. Awoyode S. (2023). LKB1 signaling and patient survival outcomes in hepatocellular carcinoma. Pharmacol. Res. 192, 106757. 10.1016/j.phrs.2023.106757 37023992

[B42] Park J. Yoon Y.-S. Han H.-S. Kim Y.-H. Ogawa Y. Park K.-G. (2014). SIK2 is critical in the regulation of lipid homeostasis and adipogenesis *in vivo* . Diabetes 63, 3659–3673. 10.2337/db13-1423 24898145

[B43] Rong Z. Zhang L. Li Z. Xiao Z. Duan Y. Ren X. (2022). Correction: SIK2 maintains breast cancer stemness by phosphorylating LRP6 and activating Wnt/β-catenin signaling. Oncogene 41, 3585. 10.1038/s41388-022-02374-y 35701535

[B44] Roos D. S. (2001). Computational biology. Bioinformatics--trying to swim in a sea of data. Science 291, 1260–1261. 10.1126/science.291.5507.1260 11233452

[B45] Schwabe R. F. Luedde T. (2018). Apoptosis and necroptosis in the liver: a matter of life and death. Nat. Rev. Gastroenterol. Hepatol. 15, 738–752. 10.1038/s41575-018-0065-y 30250076 PMC6490680

[B46] Shi X. Yu X. Wang J. Bian S. Li Q. Fu F. (2022). SIK2 promotes ovarian cancer cell motility and metastasis by phosphorylating MYLK. Mol. Oncol. 16, 2558–2574. 10.1002/1878-0261.13208 35278271 PMC9251837

[B47] Shimizu S. Kanaseki T. Mizushima N. Mizuta T. Arakawa-Kobayashi S. Thompson C. B. (2004). Role of Bcl-2 family proteins in a non-apoptotic programmed cell death dependent on autophagy genes. Nat. Cell Biol. 6, 1221–1228. 10.1038/ncb1192 15558033

[B48] Shu Z. Li X. Zhang W. Huyan Z. Cheng D. Xie S. (2024). MG-132 activates sodium palmitate-induced autophagy in human vascular smooth muscle cells and inhibits senescence *via* the PI3K/AKT/mTOR axis. Lipids Health Dis. 23, 282. 10.1186/s12944-024-02268-w 39232759 PMC11373134

[B49] Siegel R. L. Miller K. D. Wagle N. S. Jemal A. (2023). Cancer statistics, 2023. CA Cancer J. Clin. 73, 17–48. 10.3322/caac.21763 36633525

[B50] Sorice M. (2022). Crosstalk of autophagy and apoptosis. Cells 11, 1479. 10.3390/cells11091479 35563785 PMC9102887

[B51] Su C. Zhang H. Mo J. Liao Z. Zhang B. Zhu P. (2024). SP1‐activated USP27X‐AS1 promotes hepatocellular carcinoma progression *via* USP7‐mediated AKT stabilisation. Clin. and Transl. Med 14, e1563. 10.1002/ctm2.1563 38279869 PMC10819096

[B52] Sukumaran P. Nascimento Da Conceicao V. Sun Y. Ahamad N. Saraiva L. R. Selvaraj S. (2021). Calcium signaling regulates autophagy and apoptosis. Cells 10, 2125. 10.3390/cells10082125 34440894 PMC8394685

[B53] Sun Z. Jiang Q. Li J. Guo J. (2020). The potent roles of salt-inducible kinases (SIKs) in metabolic homeostasis and tumorigenesis. Sig Transduct. Target Ther. 5, 150. 10.1038/s41392-020-00265-w 32788639 PMC7423983

[B54] Thoen L. F. R. Guimarães E. L. M. Dollé L. Mannaerts I. Najimi M. Sokal E. (2011). A role for autophagy during hepatic stellate cell activation. J. Hepatology 55, 1353–1360. 10.1016/j.jhep.2011.07.010 21803012

[B55] Tsui Y.-M. Ho D. W.-H. Sze K. M.-F. Lee J. M.-F. Lee E. Zhang Q. (2024). Sorted-Cell sequencing on HCC specimens reveals EPS8L3 as a key player in CD24/CD13/EpCAM-Triple positive, stemness-related HCC cells. Cell. Mol. Gastroenterology Hepatology 18, 101358. 10.1016/j.jcmgh.2024.05.006 38750898 PMC11238133

[B56] Ullah R. Yin Q. Snell A. H. Wan L. (2022). RAF-MEK-ERK pathway in cancer evolution and treatment. Semin. Cancer Biol. 85, 123–154. 10.1016/j.semcancer.2021.05.010 33992782

[B57] Vizcaíno C. Mansilla S. Portugal J. (2015). Sp1 transcription factor: a long-standing target in cancer chemotherapy. Pharmacol. Ther. 152, 111–124. 10.1016/j.pharmthera.2015.05.008 25960131

[B58] Vogel A. Meyer T. Sapisochin G. Salem R. Saborowski A. (2022). Hepatocellular carcinoma. Lancet 400, 1345–1362. 10.1016/S0140-6736(22)01200-4 36084663

[B59] Wang H.-H. Lin C.-Y. Su S.-H. Chuang C.-T. Chang Y.-L. Lee T.-Y. (2016). Activation of salt-inducible kinase 2 promotes the viability of peritoneal mesothelial cells exposed to stress of peritoneal dialysis. Cell Death Dis. 7, e2298. 10.1038/cddis.2016.79 27441650 PMC4973365

[B60] Wang H. Liu Y. Wang D. Xu Y. Dong R. Yang Y. (2019). The upstream pathway of mTOR-Mediated autophagy in liver diseases. Cells 8, 1597. 10.3390/cells8121597 31835352 PMC6953127

[B61] Wang S. Zhu L. Li T. Lin X. Zheng Y. Xu D. (2024). Disruption of MerTK increases the efficacy of checkpoint inhibitor by enhancing ferroptosis and immune response in hepatocellular carcinoma. Cell Rep. Med. 5, 101415. 10.1016/j.xcrm.2024.101415 38382467 PMC10897610

[B62] Xiao H. Yao Z. Li T. Fang X. Xu X. Hu S. (2025). SERPINH1 secretion by cancer-associated fibroblasts promotes hepatocellular carcinoma malignancy through SENP3-mediated SP1/SQLE pathway. Int. Immunopharmacol. 150, 114259. 10.1016/j.intimp.2025.114259 39946769

[B63] Xu H. Ren D. (2015). Lysosomal physiology. Annu. Rev. Physiol. 77, 57–80. 10.1146/annurev-physiol-021014-071649 25668017 PMC4524569

[B64] Xu Y. Zhou J. Li L. Yang W. Zhang Z. Zhang K. (2022). FTO-mediated autophagy promotes progression of clear cell renal cell carcinoma *via* regulating SIK2 mRNA stability. Int. J. Biol. Sci. 18, 5943–5962. 10.7150/ijbs.77774 36263177 PMC9576516

[B65] Yang F.-C. Tan B. C.-M. Chen W.-H. Lin Y.-H. Huang J.-Y. Chang H.-Y. (2013). Reversible acetylation regulates salt-inducible kinase (SIK2) and its function in autophagy. J. Biol. Chem. 288, 6227–6237. 10.1074/jbc.M112.431239 23322770 PMC3585058

[B66] Yang Y. Wang Q. Song D. Zen R. Zhang L. Wang Y. (2020). Lysosomal dysfunction and autophagy blockade contribute to autophagy-related cancer suppressing peptide-induced cytotoxic death of cervical cancer cells through the AMPK/mTOR pathway. J. Exp. Clin. Cancer Res. 39, 197. 10.1186/s13046-020-01701-z 32962728 PMC7510096

[B67] Young M. M. Takahashi Y. Khan O. Park S. Hori T. Yun J. (2012). Autophagosomal membrane serves as platform for intracellular death-inducing signaling complex (iDISC)-mediated Caspase-8 activation and apoptosis. J. Biol. Chem. 287, 12455–12468. 10.1074/jbc.M111.309104 22362782 PMC3320995

[B68] Yu L. Alva A. Su H. Dutt P. Freundt E. Welsh S. (2004). Regulation of an ATG7-beclin 1 program of autophagic cell death by caspase-8. Science 304, 1500–1502. 10.1126/science.1096645 15131264

[B69] Yu K. Ramkumar N. Wong K. K. L. Tettweiler G. Verheyen E. M. (2023). The AMPK-Like protein kinases Sik2 and Sik3 interact with hipk and induce synergistic tumorigenesis in a drosophila cancer model. Front. Cell Dev. Biol. 11, 1214539. 10.3389/fcell.2023.1214539 37854071 PMC10579798

[B70] Yuan B.-Z. Chapman J. A. Reynolds S. H. (2008). Proteasome inhibitor MG132 induces apoptosis and inhibits invasion of human malignant pleural mesothelioma cells. Transl. Oncol. 1, 129–140. 10.1593/tlo.08133 18795123 PMC2533141

[B71] Zhang R. Liu Y. Zhong W. Hu Z. Wu C. Ma M. (2022). SIK2 improving mitochondrial autophagy restriction induced by cerebral ischemia-reperfusion in rats. Front. Pharmacol. 13, 683898. 10.3389/fphar.2022.683898 35586047 PMC9108450

[B72] Zhao Y. G. Zhang H. (2019). Core autophagy genes and human diseases. Curr. Opin. Cell Biol. 61, 117–125. 10.1016/j.ceb.2019.08.003 31480011

[B73] Zhao J. Zhang X. Gao T. Wang S. Hou Y. Yuan P. (2020). SIK2 enhances synthesis of fatty acid and cholesterol in ovarian cancer cells and tumor growth through PI3K/Akt signaling pathway. Cell Death Dis. 11, 25. 10.1038/s41419-019-2221-x 31932581 PMC6957524

[B74] Zhou J. Alfraidi A. Zhang S. Santiago-O’Farrill J. M. Yerramreddy Reddy V. K. Alsaadi A. (2017). A novel compound ARN-3236 inhibits salt-inducible kinase 2 and sensitizes ovarian cancer cell lines and xenografts to paclitaxel. Clin. Cancer Res. 23, 1945–1954. 10.1158/1078-0432.CCR-16-1562 27678456 PMC5436602

[B75] Zhu J. Li C. Wang P. Liu Y. Li Z. Chen Z. (2023). Deficiency of salt-inducible kinase 2 (SIK2) promotes immune injury by inhibiting the maturation of lymphocytes. MedComm 4 (2020), e366. 10.1002/mco2.366 37706195 PMC10495731

